# Predicting imminent risk for fracture in patients aged 50 or older with osteoporosis using US claims data

**DOI:** 10.1007/s11657-016-0280-5

**Published:** 2016-07-30

**Authors:** M. Bonafede, N. Shi, R. Barron, X. Li, D.B. Crittenden, D. Chandler

**Affiliations:** 1 Truven Health Analytics, Cambridge, MA USA; 2 Amgen Inc., One Amgen Center Drive, Thousand Oaks, CA USA

**Keywords:** Falls, Fractures, Osteoporosis, Risk factors, Insurance claims

## Abstract

**Summary:**

Patient characteristics contributing to imminent risk for fracture, defined as risk of near-term fracture within the next 12 to 24 months, have not been well defined. In patients without recent fracture, we identified factors predicting imminent risk for vertebral/nonvertebral fracture, including falls, age, comorbidities, and other potential fall risk factors.

**Purpose:**

Several factors contribute to long-term fracture risk in patients with osteoporosis, including age, bone mineral density, and fracture history. Some patients may be at imminent risk for fracture, defined here as a risk of near-term fracture within 12–24 months. Many patient characteristics contributing to imminent risk for fracture have not been well defined. This case-control study used US commercial and Medicare supplemental insured data for women and men without recent fracture to identify factors associated with imminent risk for fracture.

**Methods:**

Patients included were aged ≥50 with osteoporosis, had a vertebral or nonvertebral fracture claim (index date; fracture group) or no fracture claim (control group) from January 1, 2006, to September 30, 2012, continuously enrolled and without fracture in the 24 months before index. Potential risk factors during the period before fracture were assessed.

**Results:**

Using data from 12 months before fracture, factors significantly associated with imminent risk for fracture were previous falls, older age, poorer health status, specific comorbidities (psychosis, Alzheimer’s disease, central nervous system disease), and other fall risk factors (wheelchair use, psychoactive medication use, mobility impairment). Similar findings were observed with data from 24 months before fracture.

**Conclusions:**

In patients with osteoporosis and no recent fracture, falls, older age, poorer health status, comorbidities, and other potential fall risk factors were predictive of imminent risk for fracture. Identification of factors associated with imminent risk for vertebral/nonvertebral fracture may help identify and risk stratify those patients most in need of immediate and appropriate treatment to decrease fracture risk.

**Electronic supplementary material:**

The online version of this article (doi:10.1007/s11657-016-0280-5) contains supplementary material, which is available to authorized users.

## Introduction

Osteoporosis poses a significant and increasing health burden for the aging population [1, 2]. Osteoporotic fractures are common and associated with significant morbidity, mortality, and healthcare resource use [1, 3, 4]. Nonetheless, osteoporosis remains widely undertreated, even in patients with recent fractures [5, 6].

Several factors contribute to long-term fracture risk in patients with osteoporosis, including advanced age, fracture history, recent falls, low bone mineral density (BMD), and certain comorbidities, such as rheumatoid arthritis and causes of secondary osteoporosis [7, 8]. Risk can be quantified using fracture risk assessment tools (e.g., FRAX® and QFracture®), which estimate the 10-year risk of fracture [7, 9]. However, risk may not be constant over a 10-year period and being able to quantify vertebral or nonvertebral fracture risk over a shorter time period could assist clinicians in identifying and targeting therapy in patients with osteoporosis.

Risk factors that increase imminent risk, i.e., risk for fracture within the next 12 to 24 months, have not been well characterized. Studies suggest that after a recent fracture, the risk for a subsequent fracture is highest within the next 12 to 24 months [10–13]. Other risk factors that may not be captured in long-term fracture risk assessment tools include specific comorbidities and medications affecting blood pressure, cognitive function, and/or patient awareness that may contribute to the risk for falls, thus increasing the risk of fracture [14–18].

Using a large US commercial and Medicare supplemental claims data set, we undertook a case-control study aimed at identifying risk factors associated with imminent fracture in patients with osteoporosis who have not had a recent fracture.

## Methods

### Study design and population

This was a case-control study conducted using administrative claims data for individuals with osteoporosis who were commercially insured or who had Medicare supplemental insurance in the USA. The study objective was to identify factors predictive of imminent risk for fragility fracture, defined as a risk of near-term fracture within the next 12 to 24 months in patients with osteoporosis without a recent documented fracture (in the previous 24 months).

### Data sources

Data were obtained from the Truven Health Analytics MarketScan® Commercial Claims and Encounters (Commercial) and the Medicare Supplemental and Coordination of Benefits (Medicare) databases. The Market-Scan Commercial database contains the inpatient, outpatient, and outpatient prescription drug data of approximately 35 million employees and their dependents covered under a variety of fee-for-service and managed care plans annually. The MarketScan Medicare Supplemental and Coordination of Benefits database contains the same healthcare data for approximately 4 million retirees and/or their dependents with employer-sponsored Medicare supplemental health insurance.

All patient data used in this analysis were de-identified in compliance with the Health Insurance Portability and Accountability Act regulations; therefore, the study did not require Institutional Review Board approval.

### Patient eligibility

All patients included in the study had at least one primary or secondary diagnosis for osteoporosis (*International Classification of Diseases, Ninth Revision, Clinical Modification* [*ICD-9-CM*] diagnosis 733.0x) on an inpatient claim or an outpatient diagnosis associated with physician evaluation or management between January 1, 2004, and December 31, 2012.

Patients in the fracture group had a qualified claim for a fragility fracture between January 1, 2006, and September 30, 2012, and an osteoporosis diagnosis from January 1, 2004, to 90 days after the fracture diagnosis. Patients were identified as having a fragility fracture at hip, vertebral, or nonhip/nonvertebral sites (radius and ulna, humerus, tibia and fibula, ankle, pelvis, and clavicle) based on the presence of a primary or secondary diagnosis using *ICD-9-CM* diagnosis codes indicative of closed or pathologic fracture or an inpatient or outpatient claim that carried a diagnosis of fracture and a corresponding fracture treatment for the same fracture site. For vertebral fracture, an outpatient physician evaluation and management claim with vertebral fracture diagnosis on the same claim also qualified for inclusion. Fracture claims accompanied by any indication of major trauma (transport accidents or other causes that may imply traumatic fracture; *ICD-9-CM* diagnosis codes E800–848, E881–884, E908–909, E916–928) within 7 days before or after the fracture diagnosis were disqualified. The date of the first qualified fracture claim was set as the index date.

Patients in the control group had no claim for fracture between January 1, 2004, and December 31, 2012. An index date was randomly assigned based on the date of first osteoporosis diagnosis and the distribution of index dates in the fracture group.

Eligible patients were required to be at least 50 years of age at the index date, to be continuously enrolled for ≥730 days (24 months) before the index date (preindex period), and to have no fractures in the preindex period. Patients in both groups were excluded if any of the following conditions occurred in the 24-month preindex period: Paget disease, osteogenesis imperfecta, hypercalcemia, malignant cancer (identified by either *ICD-9-CM* diagnosis codes or chemotherapy), HIV, or preventative treatment (raloxifene) in patients with a history of breast cancer.

### Assessments

#### Fracture risk factors

More than 60 patient characteristics and potential risk factors for fracture, identified based on a literature review and clinical input, were assessed. These included demographic factors (e.g., history of falls, age, sex, geographic region, insurance plan type, the season that fracture occurred), comorbidities (e.g., Deyo-Charlson Comorbidity Index [DCI; a measure of general health] [19], central nervous system [CNS] disease, psychoses, Alzheimer’s disease), concomitant medications (e.g., number of unique medications used; use of narcotics, antidepressants, or sedatives/sleep aids), and mobility/frailty factors (e.g., wheelchair use, mobility impairment, home healthcare, being in a nursing home). The full list of potential risk factors is shown in Supplemental Table S1.

**Fig. 1:**
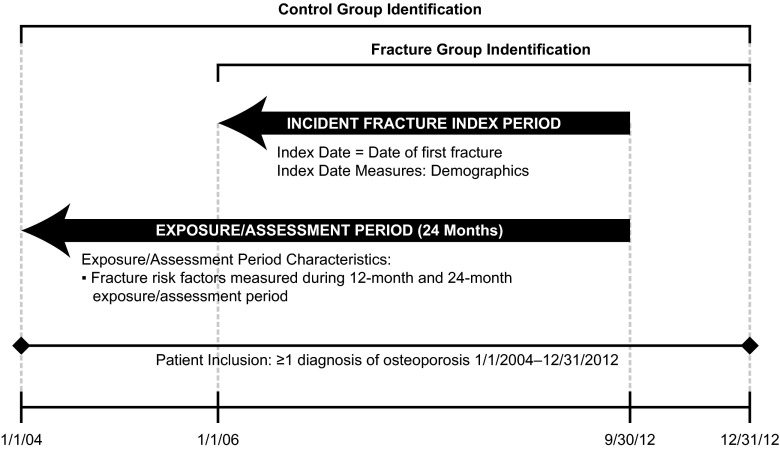
Study periods

The fracture risk factor evaluation periods are shown in Fig. 1. Unless specified, demographic variables were measured on the study index date. General health status measures were examined based on a 24-month preindex period. Clinical factors, concomitant medications, and other factors were captured during the 12- and 24-month preindex periods.

#### Statistical analysis

Descriptive analysis was conducted, including baseline and follow-up measures for all patients meeting the study criteria. Counts and proportions were provided for categorical variables, and counts, means, and standard deviations were provided for continuous variables. Risk factors for fracture were identified by multivariate logistic regression models with a binary indicator of fracture as the outcome model and covariates that included potential risk factors measured during a fixed period before the fracture date or assigned date for controls. Odds ratio of fracture was estimated for each potential risk factor and those factors with a significant odds ratio >1 were reported.

## Results

### Patients

**Fig. 2:**
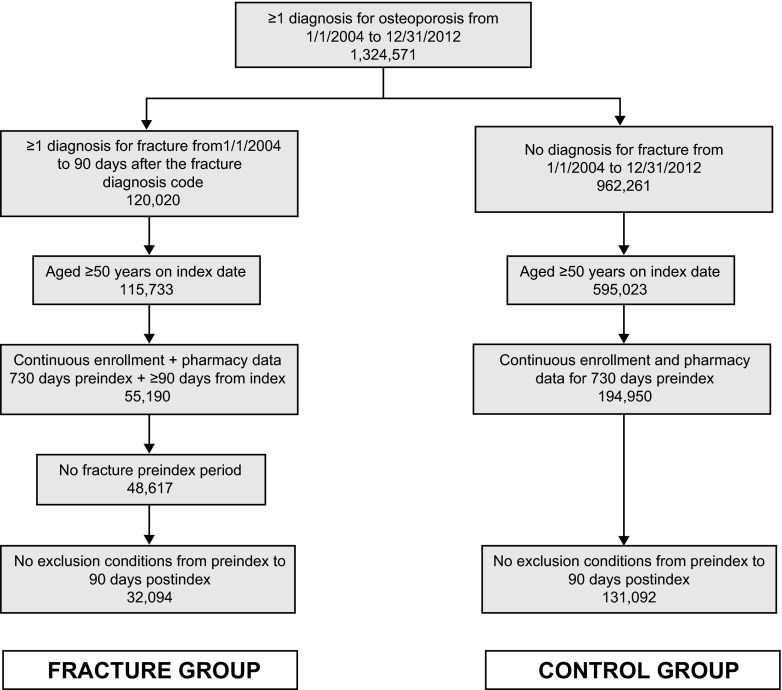
Patient counts at key points in patient data set selection

Of the 1,324,571 patients with an osteoporosis diagnosis, 163,186 met all inclusion criteria. Of these, 32,094 had a fracture and formed the fracture group and 131,092 did not have a fracture diagnosis and formed the control (nonfracture) group (Fig. 2). The study population consisted of 29,004 women and 3090 men in the fracture group and 119,922 women and 11,170 men in the control group (Table 1). The mean age of patients in the control group was lower than the fracture group. In the fracture group, fractures were primarily nonhip/nonvertebral (41.6 %), followed by vertebral (33.1 %) and hip (25.4 %).

**Table 1:** Demographic characteristics

	Fracture group	Control group
	*N* = 32,094	*N* = 131,092
Sex, *n* (%)		
Male	3090 (9.6)	11,170 (8.5)
Female	29,004 (90.4)	119,922 (91.5)
Mean (SD) age, years	75.2 (11.7)	66.4 (10.4)
US geographic region, *n* (%)		
Northeast	4277 (13.3)	19,929 (15.2)
North central	11,312 (35.2)	35,462 (27.1)
South	9954 (31.0)	46,908 (35.8)
West	6484 (20.2)	28,382 (21.7)
Unknown	67 (0.2)	411 (0.3)
Health plan type, *n* (%)		
Comprehensive	12,797 (39.9)	31,613 (24.1)
PPO	12,694 (39.6)	63,376 (48.3)
POS	1322 (4.1)	9101 (6.9)
HMO	4220 (13.1)	19,740 (15.1)
Other	382 (1.2)	3458 (2.6)
Unknown	679 (2.1)	3804 (2.9)
Index fracture type, *n* (%)		
Hip	8147 (25.4)	
Vertebral	10,608 (33.1)	
Nonhip/nonvertebral	13,339 (41.6)	

*HMO* health maintenance organization, *POS* point of service, *PPO* preferred provider organization

### Factors associated with imminent risk for fracture

The 12- and 24-month preindex predictors of imminent risk for fracture are included in Table 2. Of the factors assessed, falls were the greatest risk factor for imminent fracture within the next 12 months (odds ratio, 6.67 [95 % CI, 6.03–7.37]). Other significant risk factors included advancing age (each additional decade after the age of 50), poorer health status assessed by DCI score >0, specific comorbidities (e.g., CNS disease, psychoses, Alzheimer’s disease), concomitant medications (e.g., antidepressants [selective serotonin reuptake inhibitors (SSRIs), non-SSRIs, tricyclics], anti-Parkinson medication, tranquilizers, narcotics, sedatives/sleep aids, muscle relaxants), as well as factors related to mobility/frailty (e.g., wheelchair use, nursing home, home healthcare, mobility impairment). Similar predictors were observed for imminent risk of fracture within the next 24 months, with the exception of tricyclics (Table 2).

**Table 2:** Risk factors predictive of imminent risk for fracture (OR > 1) at any fracture site

Predictor	12 months prefracture OR (95 % CI)	*P* value	24 months prefracture OR (95 % CI)	*P* value
Demographic and general health characteristics				
History of falls	6.67 (6.03–7.37)	<0.0001	4.43 (4.09–4.80)	<0.0001
Every additional decade after age 50 years	2.00 (1.98–2.03)	<0.0001	1.97 (1.94–2.00)	<0.0001
Comorbidities				
CNS disease	1.41 (1.30–1.52)	<0.0001	1.34 (1.25–1.43)	<0.0001
Psychoses	1.37 (1.26–1.48)	<0.0001	1.34 (1.25–1.44)	<0.0001
Alzheimer’s disease	1.35 (1.22–1.50)	<0.0001	1.25 (1.14–1.37)	<0.0001
General health status measures				
DCI^a^				
≥4	1.46 (1.35–1.59)	<0.0001	1.49 (1.38–1.62)	<0.0001
3	1.40 (1.31–1.50)	<0.0001	1.42 (1.32–1.52)	<0.0001
2	1.26 (1.19–1.32)	<0.0001	1.29 (1.22–1.36)	<0.0001
1	1.16 (1.12–1.20)	<0.0001	1.17 (1.13–1.22)	<0.0001
Concomitant medications				
Narcotics	2.11 (2.05–2.18)	<0.0001	1.85 (1.79–1.92)	<0.0001
Antidepressants—SSRIs	1.47 (1.41–1.52)	<0.0001	1.41 (1.36–1.46)	<0.0001
Muscle relaxants	1.40 (1.34–1.47)	<0.0001	1.29 (1.25–1.35)	<0.0001
Tranquilizers	1.29 (1.18–1.40)	<0.0001	1.24 (1.15–1.34)	<0.0001
Antidepressants—other	1.27 (1.21–1.33)	<0.0001	1.22 (1.16–1.27)	<0.0001
Anti-Parkinson	1.24 (1.15–1.35)	<0.0001	1.20 (1.11–1.29)	<0.0001
Antidepressants—tricyclics	1.09 (1.02–1.17)	0.0119	–	NS
Sedatives and sleep aids, excluding benzodiazepines	1.05 (1.00–1.11)	0.0376	1.08 (1.03–1.12)	0.0010
Mobility/frailty				
Wheelchair use	1.79 (1.61–2.00)	<0.0001	1.80 (1.64–1.97)	<0.0001
Mobility impairment	1.46 (1.41–1.51)	<0.0001	1.39 (1.34–1.43)	<0.0001
Home healthcare	1.24 (1.16–1.34)	<0.0001	1.20 (1.13–1.28)	<0.0001
Nursing home	1.19 (1.12–1.28)	<0.0001	1.19 (1.13–1.26)	<0.0001

Risk factors with OR > 1 and significant (*P* < 0.05) included on table

*CNS* central nervous system, *DCI* Deyo-Charlson Comorbidity Index, *NS* nonsignificant, *OR* odds ratio, *SSRI* selective serotonin reuptake inhibitor

^a^Reference group: 0

Overall, risk factors were similar regardless of index fracture type (Supplemental Table S2), including falls, advancing age, and poorer health status. Although comorbidities, concomitant medications, and factors related to mobility/frailty also predicted risk for fracture across index fracture type, there were some differences in the specific diseases, medications, and mobility/fragility factors by index fracture type and time period. For instance, psychoses, Alzheimer’s disease, and tricyclics, were not identified as being predictive of fracture within the next 12 and/or 24 months for patients with a vertebral or nonhip/nonvertebral fracture but were found to be predictive for hip fracture.

## Discussion

Using a large claims database, this study identified factors contributing to imminent risk for hip vertebral, or nonvertebral fracture in patients with osteoporosis without recent fracture. Factors consistently associated with imminent risk for fracture included previous falls, advancing age, and poorer health status. Additional fall risk factors, such as specific comorbidities associated with impaired cognitive and physical function, concomitant medications, and factors related to mobility/frailty, were predictive at specific fracture sites.

This study assessed patients with osteoporosis without a recent fracture to identify clinical factors, other than recent fracture, that may contribute to imminent risk for fracture [20]; recent fracture is a recognized strong predictor of imminent fracture risk [21, 22]. Not surprisingly, falls and risk factors associated with falls were predictive of fracture in our study. Previous studies assessing imminent fracture risk and factors affecting fall risk have shown that advancing age, falls, comorbidities, and certain medications increase the risk of fracture [23–25]. Both the National Osteoporosis Foundation and the World Health Organization have identified a history of falls as a major risk for fracture [2, 26]. Indeed, it has been reported that more than 90 % of hip fractures occur following a fall [27]. In addition, the incidence of falls increases with advancing age because of age-related physical and mental changes [28]. A systematic review of observational studies on risk factors for falling in community-dwelling older people showed that certain health conditions and impairments contributed independently to the risk of falls [29]. Conditions such as stroke, dementia, depression, and Parkinson’s disease are more common in older adults and may increase fall risk due to impact on cognition and physical function [28–31]. Also, the medications used to manage these conditions can increase fall risk through their effect on cognitive and physical functioning [25, 28, 32]. For example, psychoactive medications (e.g., antidepressants, sedatives, antipsychotics), anti-Parkinson drugs, and adverse treatment events associated with these medications, including unsteadiness, impaired alertness, and dizziness, increase fall risk [15, 25, 29]. Moreover, the risk of falling increases with the number of risk factors [28].

Overall, the risk factors identified by our study for imminent risk for fracture are consistent with prior studies assessing fracture risk over longer time horizons [14–17, 33–36]. However, many of these risk factors are not accounted for in the long-term fracture risk assessment models commonly used in clinical practice. Indeed, it has been suggested that the lack of fall risk assessment with FRAX® may underestimate fracture probability, particularly in individuals with a history of falls, and inclusion could improve existing fracture prediction algorithms [37, 38].

Since older people commonly have multiple risk factors, including several comorbidities, and use multiple medications, knowledge about factors that increase risk may help guide identification, and appropriate treatment, of patients at imminent risk for fracture. Identification and management of these patients are important because of the increased probability of fracture and the associated morbidity, mortality, and cost of fracture [1, 3–5, 39, 40]. Indeed, fractures are associated with decline in functional status and health-related quality of life [39, 41], lead to the development of comorbidities [2, 42], and increase the risk of death by 25 % [3].

Management could include therapy to increase BMD and interventions to increase bone strength, in addition to interventions to reduce falls. Although fall prevention strategies and interventions to decrease and/or protect from falls could be beneficial in reducing fracture, these options have had limited success and clinical studies have not consistently shown reductions in the risk of falling [43, 44]. Other management options may be required to strengthen bone and thus reduce the likelihood of fracture if a fall occurs.

Strengths of this study include the large study population over a time period of 2004 to 2012, with patients from across the USA and various types of health plans. This study had several limitations, including the potential for underreporting of falls and fractures; only falls resulting in medical events, procedures, and treatment were coded and included, and this may not be a reliable measure of frequency. We could not positively identify fragility fractures from medical claims based on ICD-9-CM diagnosis codes; therefore, we required closed or pathologic fracture in a specific treatment setting and/or accompanied by physician evaluation and management code, and E-codes to rule out fracture due to trauma. As a result, imminent risk may be underestimated in this study. Parental fracture history and BMD data, which are known risk factors for fractures, were unavailable from administrative claims data, limiting the number of risk factors examined. In addition, all comorbidities may not be captured by claims coding or may be miscoded. Owing to the smaller sample of males included in this study, analysis by sex was not undertaken, and thus sex differences could not be assessed. Finally, this population of patients who were either commercially insured or who had Medicare supplemental health insurance may not be representative of other insured or uninsured patient groups.

In conclusion, in men and women with osteoporosis and no recent fracture, multiple factors were associated with imminent risk for fracture, including older age, falls, comorbidities affecting cognitive or physical function, and other potential fall risk factors. Identification of factors putting patients at imminent risk for fracture may help risk stratify those patients most at need of immediate and appropriate treatment to decrease fracture risk.

## Electronic supplementary material

Below is the link to the electronic supplementary material.

ESM 1 Table S1 Fracture Risk Factors Assessed. (DOCX 17 kb)

ESM 2 Table S2 Predictors of Imminent Risk for Fracture (OR >1) by Fracture Location. (DOCX 32.4 kb)
