# Supplementary material for: Predicting imminent risk for fracture in patients aged 50 or older with osteoporosis using US claims data
Source: Arch Osteoporos. 2016 Jul 30;11(1):26. doi: 10.1007/s11657-016-0280-5 (PMC4967418; doi:10.1007/s11657-016-0280-5)
Supplement: Supplementary file 1 — Table S1 Fracture Risk Factors Assessed. (DOCX 17 kb) [file 11657_2016_280_MOESM1_ESM.docx]

**Supplemental Table S1. Fracture Risk Factors Assessed**

| Demographic factors and general health characteristics | |
| --- | --- |
| - History of falls - Age - Geographic region - Index year | - Sex - Insurance plan type - Season of fracture - BMD tests |
| Comorbidities | |
| - DCI (measure of general health) - Dementia - Psychoses^a^ - Depression - Mood disorders (anxiety disorders, bipolar disorder) - Alzheimer’s disease - CNS disease (eg, multiple sclerosis, Parkinson’s disease) - Cardiovascular disease (diagnosis-based) - Cardiovascular procedure - Stroke - Other cerebrovascular events (excluding stroke) - Venous thromboembolism - Diabetes | - Blood pressure abnormalities - Osteoarthritis - Vision impairments - Mobility impairment - Vertigo/dizziness - Smoking - Alcohol abuse/substance abuse - Inflammatory bowel disease (Crohn disease and ulcerative colitis) - Thyroid disorders (untreated hyperthyroidism, overtreated hypothyroidism) - Chronic malnutrition/malabsorption - Hypogonadism - Rheumatoid arthritis - Chronic liver disease |
| Concomitant medications | |
| - Antidepressants (tricyclics) - Antidepressants (others) - Tranquilizers - Sedatives/sleep aids (excluding benzodiazepines) - Benzodiazepines - Anti-Parkinson drugs - Asthma/COPD agents - Antidiarrheals - Antiemetics - Inflammatory bowel agents - Other anticholinergics - Antihistamines - Number of unique medications | - Antidepressants (SSRIs) - Narcotics - Muscle relaxants - Corticosteroids - NSAIDs - Hypoglycemics - Histamine-2 blockers - Cardiovascular drugs (excluding antihypertensives) - Antihypertensives - Anticoagulants - Rehydration agents - DMARDs - Osteoporosis medications |
| Other factors | |
| - Durable medical equipment use^b^ - Home healthcare services | - Physical/occupational therapy - Nursing home services |

BMD=bone mineral density; CNS=central nervous system; COPD=chronic obstructive pulmonary disease; DCI=Deyo-Charlson Comorbidity Index; DMARD=disease-modifying antirheumatic drug; NSAID=nonsteroidal anti-inflammatory drug; SSRI=selective serotonin reuptake inhibitor.

^a^Includes alcohol-induced mental disorders, drug-induced mental disorders, transient mental disorders, persistent mental disorders, schizophrenia, and delusional disorders.

^b^Includes crutch/walker/cane, wheelchair, oxygen use, hospital bed, transfer bench, mattress.
