# Supplementary material for: Predicting imminent risk for fracture in patients aged 50 or older with osteoporosis using US claims data
Source: Arch Osteoporos. 2016 Jul 30;11(1):26. doi: 10.1007/s11657-016-0280-5 (PMC4967418; doi:10.1007/s11657-016-0280-5)
Supplement: Supplementary file 2 — Table S2 Predictors of Imminent Risk for Fracture (OR >1) by Fracture Location. (DOCX 32.4 kb) [file 11657_2016_280_MOESM2_ESM.docx]

**Supplemental Table S2. Predictors of Imminent Risk for Fracture (OR >1) by Fracture Location**

| **Predictor** | **12 Months Prefracture, OR (95% CI)** | ***P* Value** | **24 Months Prefracture, OR (95% CI)** | ***P* Value** |
| --- | --- | --- | --- | --- |
| **Hip fracture** | | | | |
| Demographic and general health characteristics | | | | |
| History of falls | 1.57 (1.38–1.79) | <0.0001 | 1.54 (1.38–1.73) | <0.0001 |
| Every additional decade after age 50 years | 2.22 (2.17–2.28) | <0.0001 | 2.18 (2.13–2.23) | <0.0001 |
|  |  |  |  |  |
| Comorbidities |  |  |  |  |
| CNS disease | 1.34 (1.20–1.50) | <0.0001 | 1.35 (1.22–1.49) | <0.0001 |
| Psychoses | 1.35 (1.21–1.49) | <0.0001 | 1.40 (1.27–1.54) | <0.0001 |
| Alzheimer’s disease | 1.37 (1.21–1.55) | <0.0001 | 1.37 (1.23–1.54) | <0.0001 |
|  |  |  |  |  |
| General health status measures | |  |  |  |
| DCI^a^ |  |  |  |  |
| ≥4 | 1.61 (1.42–1.82) | <0.0001 | 1.67 (1.47–1.90) | <0.0001 |
| 3 | 1.44 (1.29–1.61) | <0.0001 | 1.47 (1.32–1.65) | <0.0001 |
| 2 | 1.28 (1.18–1.40) | <0.0001 | 1.31 (1.20–1.43) | <0.0001 |
| 1 | 1.23 (1.16–1.31) | <0.0001 | 1.25 (1.17–1.33) | <0.0001 |
|  |  |  |  |  |
| Concomitant medications | | | | |
| Narcotics | 1.11 (1.05–1.17) | 0.0004 | 1.11 (1.05–1.17) | 0.0003 |
| Antidepressants–SSRIs | 1.27 (1.19–1.35) | <0.0001 | 1.24 (1.16–1.31) | <0.0001 |
| Tranquilizers | 1.46 (1.30–1.64) | <0.0001 | 1.33 (1.19–1.48) | <0.0001 |
| Antidepressants–other | 1.26 (1.17–1.36) | <0.0001 | 1.21 (1.12–1.30) | <0.0001 |
| Anti-Parkinson | 1.28 (1.14–1.45) | <0.0001 | 1.24 (1.11–1.39) | 0.0001 |
| Antidepressants–tricyclics | 1.27 (1.13–1.42) | <0.0001 | 1.19 (1.08–1.32) | 0.0005 |
| Sedatives and sleep aids, excluding benzodiazepines | 1.10 (1.01–1.20) | 0.0251 | --- | NS |
| Benzodiazepines | 1.14 (1.07–1.21) | <0.0001 | 1.10 (1.04–1.16) | 0.0013 |
|  |  |  |  |  |
| Mobility/frailty | |  |  |  |
| Wheelchair use | 1.38 (1.20–1.59) | <0.0001 | 1.32 (1.17–1.49) | <0.0001 |
| Mobility impairment | 1.59 (1.51–1.68) | <0.0001 | 1.54 (1.46–1.63) | <0.0001 |
| Home healthcare | 1.14 (1.03–1.27) | 0.0128 | 1.15 (1.05–1.26) | 0.0021 |
| Nursing home | 1.25 (1.15–1.37) | <0.0001 | 1.22 (1.13–1.32) | <0.0001 |
| **Vertebral fracture** | | | | |
| Demographic and general health characteristics | | | | |
| History of falls | 1.20 (1.06–1.36) | 0.0043 | 1.22 (1.10–1.36) | 0.0003 |
| Every additional decade after age 50 years | 2.03 (1.99–2.08) | <0.0001 | 2.00 (1.96–2.05) | <0.0001 |
| Male sex^b^ | 1.74 (1.63–1.84) | <0.0001 | 1.73 (1.62–1.83) | <0.0001 |
|  |  |  |  |  |
| Comorbidities |  |  |  |  |
| CNS disease | 1.17 (1.06–1.30) | 0.0027 | 1.15 (1.05–1.26) | 0.0034 |
|  |  |  |  |  |
| General health status measures | |  |  |  |
| DCI^a^ |  |  |  |  |
| ≥4 | 1.32 (1.18–1.47) | <0.0001 | 1.34 (1.19–1.50) | <0.0001 |
| 3 | 1.34 (1.22–1.48) | <0.0001 | 1.37 (1.24–1.51) | <0.0001 |
| 2 | 1.31 (1.22–1.41) | <0.0001 | 1.35 (1.25–1.45) | <0.0001 |
| 1 | 1.20 (1.13–1.26) | <0.0001 | 1.22 (1.15–1.29) | <0.0001 |
|  |  |  |  |  |
| Concomitant medications | | | | |
| Narcotics | 2.32 (2.20–2.44) | <0.0001 | 2.08 (1.97–2.19) | <0.0001 |
| Antidepressants–SSRIs | 1.18 (1.12–1.25) | <0.0001 | 1.17 (1.11–1.23) | <0.0001 |
| Muscle relaxants | 2.38 (2.25–2.52) | <0.0001 | 2.04 (1.93–2.15) | <0.0001 |
| Antidepressants–other | 1.07 (1.00–1.15) | 0.0445 | 1.08 (1.01–1.15) | 0.0182 |
| Sedatives and sleep aids, excluding benzodiazepines | --- | NS | 1.08 (1.01–1.14) | 0.0228 |
| Benzodiazepines | 1.06 (1.00–1.11) | 0.0352 | --- | NS |
| Corticosteroids | 1.15 (1.10–1.21) | <0.0001 | 1.05 (1.00–1.10) | 0.0477 |
|  | |  |  |  |
| Mobility/frailty | |  |  |  |
| Wheelchair use | 1.15 (1.01–1.32) | 0.0356 | 1.20 (1.07–1.35) | 0.0017 |
| Home healthcare | 1.18 (1.08–1.30) | 0.0005 | 1.10 (1.01–1.19) | 0.0262 |
| **Nonhip/Nonvertebral fracture** | | | | |
| Demographic and general health characteristics | | | | |
| History of falls | 5.39 (4.93−5.89) | <0.0001 | 4.05 (3.74–4.38) | <0.0001 |
| Every additional decade after age 50 years | 1.32 (1.30–1.35) | <0.0001 | 1.32 (1.29–1.34) | <0.0001 |
|  |  |  |  |  |
| Comorbidities |  |  |  |  |
| CNS disease | 1.27 (1.15–1.40) | <0.0001 | 1.17 (1.08−1.28) | 0.0003 |
| Diabetes | 1.10 (1.03–1.17) | 0.0053 | --- | NS |
|  | |  |  |  |
| General health status measures | |  |  |  |
| DCI^a^ |  |  |  |  |
| ≥4 | 1.14 (1.02–1.27) | 0.0165 | 1.16 (1.04–1.30) | 0.0086 |
| 3 | 1.15 (1.05–1.26) | 0.0037 | 1.15 (1.05–1.27) | 0.0033 |
| 2 | 1.08 (1.00–1.16) | 0.0366 | 1.09 (1.02–1.18) | 0.0150 |
| 1 | --- | NS | 1.05 (1.00–1.10) | 0.0483 |
| Osteoporosis medication | 1.06 (1.01–1.10) | 0.0114 | 1.10 (1.06–1.15) | <0.0001 |
|  |  |  |  |  |
| Concomitant medications | | | | |
| Narcotics | 1.98 (1.89–2.06) | <0.0001 | 1.74 (1.66–1.82) | <0.0001 |
| Antidepressants–SSRIs | 1.47 (1.40–1.55) | <0.0001 | 1.43 (1.36–1.49) | <0.0001 |
| Tranquilizers | 1.15 (1.03–1.28) | 0.0106 | 1.11 (1.01–1.23) | 0.0367 |
| Antidepressants–other | 1.27 (1.20–1.35) | <0.0001 | 1.21 (1.14–1.28) | <0.0001 |
| Anti-Parkinson | 1.13 (1.02–1.25) | 0.0254 | 1.10 (1.00–1.22) | 0.0470 |
|  | |  |  |  |
| Mobility/frailty | |  |  |  |
| Wheelchair use | 1.45 (1.28–1.65) | <0.0001 | 1.51 (1.35–1.68) | <0.0001 |
| Mobility impairment | 1.52 (1.45–1.58) | <0.0001 | 1.42 (1.36–1.48) | <0.0001 |
| Home healthcare | --- | NS | 1.09 (1.01–1.18) | 0.0294 |

CNS=central nervous system; DCI=Deyo-Charlson Comorbidity Index; NS=nonsignificant; OR=odds ratio; SSRI=selective serotonin reuptake inhibitor.

^a^Reference group: 0. ^b^Reference group: female.

Risk factors with OR >1 and significant (P<0.05) included on table.
